# Cell-Cell Adhesion as a Double-Edged Sword in Tissue Fluidity

**Published:** 2026-03-05

**Authors:** Anh Q. Nguyen, Pradip K. Bera, Jacob Notbohm, Dapeng Bi

**Affiliations:** 1 Department of Physics, Northeastern University, Boston, MA 02115, USA; 2 Center for Theoretical Biological Physics, Northeastern University, Boston, MA 02115, USA; 3 Department of Mechanical Engineering, University of Wisconsin–Madison, WI, USA

## Abstract

Cell migration plays a fundamental role in numerous physiological processes, including embryonic development, wound healing, and cancer metastasis. While cell–cell adhesion is known to regulate motion by shaping cell morphology and intercellular force balance, its dynamic, rate-dependent contributions to tissue behavior remain poorly understood. In this study, we examine how the dissipative nature of cell–cell adhesion influences tissue dynamics and collective migration using an extended vertex model with explicit junctional viscosity. Our findings reveal a nontrivial interplay between two distinct components of adhesion: an interfacial adhesion energy (energetic, rate-independent) contribution, which sets the effective junctional tension, and a dissipative (rate-dependent) contribution, which controls resistance to relative motion during cell rearrangements. We show that increasing the energetic component promotes migration by modifying cell shape and lowering the barrier to neighbor exchanges, whereas strengthening the dissipative component induces jamming and suppresses cell motion. Linear rheological analysis further demonstrates that, in the unjammed regime, vertex-model tissues exhibit power-law viscoelastic behavior, with adhesion modulating the power-law exponent and thereby controlling the spread of relaxation timescales. Together, these findings clarify the dual role of adhesion in governing tissue mechanics and rheology and provide a mechanistic framework for understanding the balance between fluidity and rigidity in epithelial monolayers.

## I. INTRODUCTION

Cell migration and tissue dynamics underpin a wide range of biological processes, from embryonic development and morphogenesis [1–5] to tissue repair [6–9] and cancer metastasis [10–12]. A key regulator of tissue dynamics is cell–cell adhesion, which not only maintains tissue’s mechanical integrity [13] but also mediates force transmission across cells [14, 15]. The mechanical contribution of cell–cell adhesion can be understood through two complementary aspects: the interfacial adhesion energy (energetic) and the viscous-dissipative. From the interfacial energy perspective, intercellular adhesion contributes a negative term to the effective junctional tension, thereby reducing net contractile stress along cell–cell interfaces and increasing the cell shape index [16–19]. Because higher intercellular tension renders tissues more solid-like and suppresses cellular rearrangements [20], the adhesion-induced reduction in tension effectively promotes tissue fluidity by softening cells and lowering the energy barrier for rearrangement. From the viscous-dissipative perspective, adhesion introduces resistance to relative cell motion, acting as a source of mechanical dissipation and making intercellular interactions more viscous-like [21–23].

In this context, cell–cell adhesion is often metaphorically described as a “biological glue” and is widely believed to enhance tissue solidity[24]. This view has motivated the development of jamming phase diagrams inspired by adhesive colloidal particles [25], where increasing adhesion drives the system toward a more jammed, solid-like state [26]. Consistent with this view, an in vitro study has shown that the migration of human bronchial epithelial cells (HBECs) slows down as cell–cell junctions mature, implying increased adhesive strength [27], supporting the viscous-dissipative picture. However, conflicting observations have emerged. For instance, unjamming of 4T1 epithelial monolayers under vertical compression was accompanied by increased cell–cell adhesion, as indicated by elevated cadherin density at junctions [28]. This finding is consistent with predictions from the conventional vertex model (VM), which captures the energetic aspect of cell–cell adhesion by modeling it as a rate-independent force that contributes to junctional tension [29].

In this framework, adhesion regulates cell shape and promotes neighbor exchanges by modulating intercellular mechanical tension [30, 31]. Consequently, increased adhesion can facilitate cell rearrangements and drive tissue unjamming. Notably, this interplay between cell shape and unjamming predicted by the vertex model has been observed experimentally in vitro, particularly in studies of asthmatic airway epithelium where increased cell elongation and rearrangement correlate with unjamming transitions [32]. Another example supporting this picture comes from studies of RAB5A. Elevated levels of this protein can unjam initially kinetically arrested confluent epithelial monolayers [33] and fluidize epithelial spheroids [34]. Notably, the RAB5A induced unjamming transition in confluent monolayer was accompanied by increased junctional tension, cell-cell contacts, monolayer rigidity, and E-cadherins expression [33]. The effect of RAB5A is mediated through its ability to accelerate endocytosis, which increases the endo/exocytic trafficking of cadherins from the plasma membrane, effectively enhancing E-cad turnover and cell adhesion [33, 35]. This outcome partly agrees with vertex model predictions, since increasing adhesion is expected to promote cell-cell contacts and unjamming. However, the vertex model would predict a reduction in junctional tension as adhesion rises, whereas the RAB5A experiments report the opposite. This discrepancy highlights a limitation of the conventional vertex framework, suggesting that adhesion dynamics and turnover may influence tissue mechanics in ways that go beyond a simple energetic contribution to junctional tension.

While the rate-independent (energetic) contribution of cell–cell adhesion has been extensively studied, its rate-dependent (dissipative) effects on tissue dynamics remain underexplored. In this work, we address the apparent paradox between increased adhesion and unjamming by extending the classical vertex model to include the dissipative component of cell–cell adhesion. This allows us to investigate how dynamic adhesion affects tissue migration and mechanics. Our results reveal a nontrivial interplay between the two aspects of cell-cell adhesion. Specifically, increasing the energetic component facilitates cell rearrangements and enhances collective migration, whereas enhancing the dissipative component impedes motion, leading to kinetic arrest and jamming. By explicitly separating the two complementary contributions of cell-cell adhesion, the extended vertex model provides a direct theoretical framework to dissect how adhesion regulates tissue unjamming and fluidization. We apply this framework to MDCK monolayers [36], where it quantitatively captures the experimentally observed, shape-independent changes in tissue fluidity following perturbations of cell–cell adhesion. Notably, adhesion is modulated while other key factors—including cell density, traction forces, intercellular tension, and cell shape—remain unchanged. Model–experiment comparisons indicate that the observed variations in fluidity arise predominantly from changes in the dissipative component of adhesion rather than from its energetic contribution. Beyond MDCK monolayers, our extended VM was applied to analyze morphogenetic tissue flows in the *Drosophila* embryo [37]. The model qualitatively captures the non-monotonic changes in cell shape and rearrangement rates observed when cell–cell adhesion is increased.

To further elucidate how viscous adhesion influences tissue-scale mechanical properties, we analyze the linear rheological response of the model under step and oscillatory shear. In the unjammed regime, the tissue behaves as a power-law viscoelastic fluid, agreeing with the relaxation of MDCK monolayer [38, 39]. These results establish a mechanistic link between adhesive dynamics at the cellular level and emergent tissue rheology. Together with prior studies that have focused on the energetic aspects of adhesion [40–43], our work helps build a more complete picture of how adhesion regulates tissue rheology.

## II. RESULTS

### A microscopic model of cell-cell adhesion force

A.

To incorporate a mechanically plausible yet computationally tractable representation of dissipative cell–cell adhesion into the vertex model, we began from a minimal microscopic picture based on receptor–ligand binding kinetics. In this picture, cell–cell friction arises from the formation and rupture of transient adhesion bonds between receptors and ligands distributed on opposing cell membranes. In confluent epithelial tissues, the small inter-membrane separation permits direct molecular interactions across neighboring cell surfaces, enabling such transient adhesive coupling to generate dissipative forces. As two adjacent cells move relative to one another, adhesion bonds are continually broken and reformed due to the dynamic contact between their membranes. Each time a ligand on one cell membrane encounters a receptor on the neighboring membrane, a bond may form if the interaction persists for a sufficient duration. As the relative velocity *v* between adjacent cell surfaces increases, ligand–receptor encounters occur more frequently due to the elevated collision rate. However, the contact duration of each encounter correspondingly decreases, reducing the time available for bond formation. Under these assumptions, Chang and Hammer constructed a convection-diffusion equation that describes the dynamics of ligand-receptor interactions and derived an effective encounter rate *k*_0_ [44]:

(1) $$k_{0}=2\pi D\left[\frac{I_{0}\left(Pe/2\right)}{K_{0}\left(Pe/2\right)}+2\sum_{n=1}^{\infty }{\left(-1\right)}^{n}\frac{I_{n}\left(Pe/2\right)}{K_{n}\left(Pe/2\right)}\right]$$

where D is the diffusion coefficient of adhesion protein molecules (ligands and receptors), $I_{n}$ and $K_{n}$ are modified Bessel functions of the second kind, and Pe=va/D is the Peclet number, where a is the reactive radius. Because not every encounter results in binding, the effective binding rate also depends on the probability P that a receptor-ligand pair will successfully react. Assuming the receptor can occupy any position within the reactive circle (r≤a) with equal likelihood, P represents the chance that binding occurs before the ligand exits the interaction zone. Since the diffusion coefficient of E-Cadherin in the membrane is on the order of 3 × 10^−14^ *m*^2^/*s* [45], while the separation between cell membranes in confluent tissues is on the order of 10 *nm* and the typical cell speed is 1μm/min [46, 47], we focus on the low Pe regime. In this regime, $P∼\frac{a^{2}k_{in}}{a^{2}k_{in}+8D}$, where $k_{in}$ is the intrinsic binding rate [44]. Additionally, in the low Pe limit, the encounter rate is well approximated by a linear function of Pe. The effective binding rate can be approximated by: $k_{f}=k_{0}P=\pi Dm\frac{a^{2}k_{in}}{a^{2}k_{in}+8D}Pe$, with m represents the proportional constant between $k_{0}$ and Pe in the low Pe limit. The number of adhesion bonds N between the two cell membranes evolves according to the binding–unbinding kinetics equation, $\frac{dN}{dt}=k_{f}\rho l-k_{b}N$, where ρ is the adhesion protein density per unit length, l is the length of the contact interface, and $k_{b}$ is the unbinding rate, treated here as a constant. Assuming that each bond generates a constant force f, in steady state, the cell-cell frictional adhesion force can be expressed as:

(2) $$F_{\mathrm{fric}}=fN_{\mathrm{steady}}=\frac{\pi \rho mf}{k_{b}}\frac{a^{3}k_{in}}{a^{2}k_{in}+8D}lv$$

Since the force is proportional to the relative velocity, it behaves similarly to a viscous force that discourages relative motion between adjacent membranes.

### B. Extended Vertex Model with Intercellular Adhesive Friction

To understand the influence of intercellular adhesion on tissue dynamics, we study the 2D Vertex model in which the biomechanical interaction is governed by the energy functional [29, 30]: $ℰ=\sum_{c=1}^{N}\left[K_{A}{\left(A_{c}-A_{0}\right)}^{2}+K_{P}{\left(P_{c}-P_{0}\right)}^{2}\right]$, where N is the total number of cells, $K_{A}$ and $K_{P}$ represent the area and perimeter moduli, $A_{c}$ and $P_{c}$ are the area and perimeter of cell c, and $A_{0}$ and $P_{0}$ are the preferred area and perimeter, which we treat as homogeneous across cells. To implement the intercellular adhesion force into the vertex model, we introduce a model parameter called linear damping coefficient $\xi =\frac{\pi \rho mf}{k_{b}}\frac{a^{3}k_{in}}{a^{2}k_{in}+8D}$; representing the cell-cell adhesion drag force per unit length per unit velocity. In the 2D vertex model, the contact interface between two adjacent cells is represented by the edge shared by the two polygons. Since the adhesion force on a shared edge depends on the edge length and the relative velocity between the two interfaces, we model the force in the vertex model framework as: $F_{ij}^{\text{ad}}=-\xi \left(r_{i}-r_{j}\right)\cdot \left(v_{i}-v_{j}\right){\hat{r}}_{ij}$, where $F_{ij}^{\text{ad}}$ represents the adhesion force acting on vertex i due to its relative motion with neighboring vertex $j,r_{i}$ and $v_{i}$ are the position and velocity vectors of vertex i, respectively, and ${\hat{r}}_{ij}$ is the unit vector pointing from vertex j to vertex i. This force acts as a viscous damping mechanism that resists changes in the length of the cell-cell contact interface, effectively modeling the frictional resistance arising from dynamic adhesion between adjacent cell membranes [22, 48]. The introduction of this adhesion force gives rise to the overdamped equation of motion:

(3) $$\mu {\dot{r}}_{i}+\xi \sum_{j\in S_{i}}\left[\left(r_{i}-r_{j}\right)\cdot \left({\dot{r}}_{i}-{\dot{r}}_{j}\right)\frac{r_{i}-r_{j}}{\left|r_{i}-r_{j}\right|}\right]\;\;=-\nabla_{r_{i}}ℰ+\mu v_{0}{\hat{n}}_{i}$$

where μ is the vertex-substrate viscous coefficient, $S_{i}$ represents the set of adjacent vertices to vertex $i,v_{0}$ is the propulsion strength and ${\overset{ˆ}{n}}_{i}=\left(cos\;\theta_{i},sin\;\theta_{i}\right)$ is the cell polarity, which follows a random white noise rotational diffusion with mean 0 and variance $2D_{r}$. The model can be made dimensionless by expressing all lengths and time in units of $\sqrt{A_{0}}$ and $\mu /A_{0}K_{A}$, respectively, resulting in a dimensionless energy functional: $e=\sum {\left(a_{c}-1\right)}^{2}+\kappa {\left(p_{c}-p_{0}\right)}^{2}$, where $a_{c}=A_{c}/A_{0}$, $p_{c}=P_{c}/\sqrt{A_{0}},p_{0}=P_{0}/\sqrt{A_{0}}$, and $\kappa =K_{P}/K_{A}A_{0}$. The nondimensionalized equation of motion is:

(4) $${\dot{r}}_{i}+\xi_{0}\sum_{j\in S_{i}}\left[\left(r_{ij}\cdot {\dot{r}}_{ij}\right){\hat{r}}_{ij}\right]=f_{i}+v_{0}{\overset{ˆ}{n}}_{i}$$

where $r_{ij}=r_{i}-r_{j}$ represents the edge vector from vertex j to vertex $i,f_{i}=-\nabla_{r_{i}}e$ is the nondimensionalized interaction force on vertex i, and $\xi_{0}=\frac{\xi \sqrt{A_{0}}}{\mu }$ is the dimensionless cell-cell adhesion coefficient. Similar phenomenological forms of internal viscous dissipation have recently been introduced to vertex models to study active nematics and sustained flows [48]. With the introduction of this adhesion force into the vertex model, the influence of cell–cell adhesion on tissue dynamics is captured through two complementary aspects: the energetic aspect, reflected in the shape index $p_{0}$, and the kinetic aspect, reflected in $\xi_{0}$. In the simulations used for this work, the following parameters are fixed: N=400,κ=1, and $D_{r}A_{0}K_{A}/\mu =0.5$.

### C. The dual role of cell-cell adhesion in tissue dynamics

#### Adhesion-Regulated Glassy Dynamics in Simulated Epithelial Monolayers

**FIG. 2.**
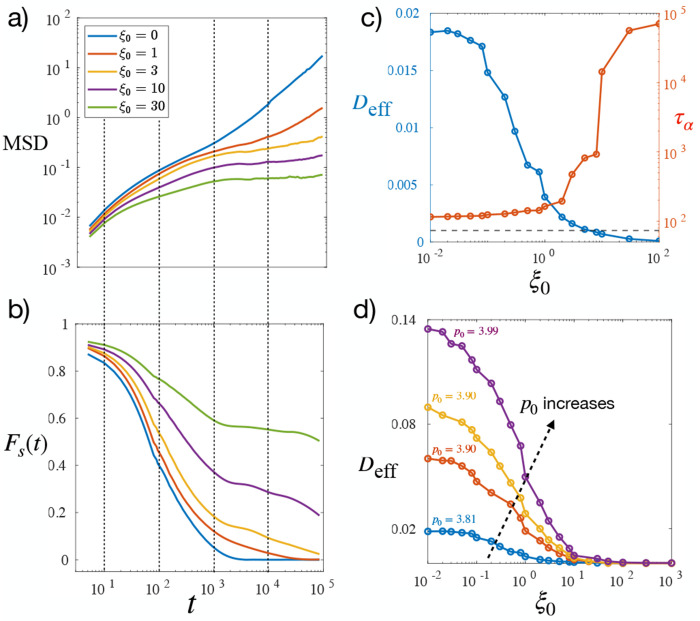
Glassy dynamics analysis: (a) Mean squared displacement (MSD) for $p_{0}=3.81$ and $v_{0}=0.05$, showing suppressed long-time diffusion with increasing $\xi_{0}$. (b) Self-intermediate scattering function $F_{s}(k,t)$ at the same parameters as in panel (a), evaluated at $k=\pi /\sqrt{A_{0}}$, illustrating the extension of the plateau and increase in relaxation time. (c) Effective diffusivity $D_{\text{eff}}$ and α-relaxation time $\tau_{\alpha }$ as functions of $\xi_{0}$, obtained from panel (a) and (b), highlight the approach to a jammed state. (d) Dependence of $D_{\text{eff}}$ on $\xi_{0}$ and $p_{0}$ while keeping $v_{0}=0.05$, demonstrating how both energetic and viscous adhesion mechanisms influence tissue dynamics (bottom to top: $p_{0}=3.81,3.87,3.9,3.99$).

While the energetic aspect of cell–cell adhesion has been extensively studied in the context of tissue dynamics and jamming transitions—both theoretically [30, 31, 49, 50] and experimentally [28, 51–53]—the kinetic (viscous-dissipative) contribution remains comparatively underexplored [22, 24, 48]. To systematically investigate its role, we examine the model at fixed values of $p_{0}=3.81$ and $v_{0}=0.05$. The mean squared displacement (MSD) as a function of time for varying levels of kinetic adhesion strength $\xi_{0}$ is shown in Fig. 2a. As $\xi_{0}$ increases, the long-time diffusive behavior of the MSD is progressively suppressed, and the intermediate-time plateau becomes more prominent and extended. This indicates stronger caging of cells by their neighbors and a shift toward glassier dynamics, where cells remain trapped in their local environment for longer times. To quantify the transition between fluid-like and jammed behavior, we use a dynamical order parameter based on the long-time self-diffusivity, defined as $D_{s}={lim}_{t\to \infty }MSD(t)/(4t)$. From this, the dimensionless effective diffusivity $D_{\text{eff}}=2D_{s}D_{r}/v_{0}^{2}$ [31] is calculated. Fig.2c shows how $D_{\text{eff}}$ varies with $\xi_{0}$. As $\xi_{0}$ increases, $D_{\text{eff}}$ decreases from a finite value to the numerical noise floor, signaling a transition from an unjammed, motile state to a jammed, arrested state driven by viscous cell-cell adhesion. Although the adhesion force in our model does not directly suppress cell motion, increasing $\xi_{0}$ effectively resists motion by penalizing changes in cell–cell junction geometry. This restricts cell rearrangement and thereby reduces large-scale tissue flow. Such adhesion-driven arrest captures the jamming behavior associated with adherens junction maturation observed in [27].

Another standard quantity to characterize glassy dynamics is self-intermediate scattering function [54], defined as $F_{s}(k,t)=\left\langle e^{ik\cdot \Delta r(t)}\right\rangle$, with ⟨…⟩ represents the temporal and angles average. Because the exponential is generally complex, the real-valued function reported in this work is obtained by taking the real part of the exponential before averaging. Fig.2b shows the relaxation of $F_{s}(t)$ at $k=\pi /\sqrt{A_{0}}$, the length scale of one cell size, representing the intrinsic cage of each cell due to their neighbors. The relaxation time $\tau_{\alpha }$ of $F_{s}$ therefore represent the timescale at which cells become uncaged and rearrange with their neighbors. Here, $\tau_{\alpha }$ was obtained by finding the time at which $F_{s}(t)$ decay to 1/e. As $\xi_{0}$ increases, the system becomes more jammed, indicated by the longer relaxation time $\tau_{\alpha }$, with $\tau_{\alpha }$ eventually exceeds the simulation time, indicated by the plateau in $F_{s}(t)$. The dependence of $\tau_{\alpha }$ on $\xi_{0}$ is presented in Fig.2c. Opposite to $D_{\text{eff}},\tau_{\alpha }$ increases monotonically as $\xi_{0}$ increases, eventually diverges as the system approaches the jamming transition. The influence of the viscous component of cell–cell adhesion, characterized by $\xi_{0}$, on tissue dynamics can be integrated with the well-established role of the energetic component, governed by $p_{0}$, which has been extensively explored in previous studies [31, 32, 55, 56]. Together, these two aspects provide a more comprehensive understanding of how cell–cell adhesion modulates tissue dynamics. As illustrated in Fig.2d, increasing viscous adhesion (higher $\xi_{0}$) leads to enhanced jamming, consistent with stronger dissipative interactions that restrict cell rearrangements. In contrast, increasing energetic adhesion (higher $p_{0}$) promotes tissue fluidization by reducing junctional tension and facilitating cell neighbor exchanges [30].

#### Adhesion-Driven Jamming Phase Diagram

**FIG. 3.**
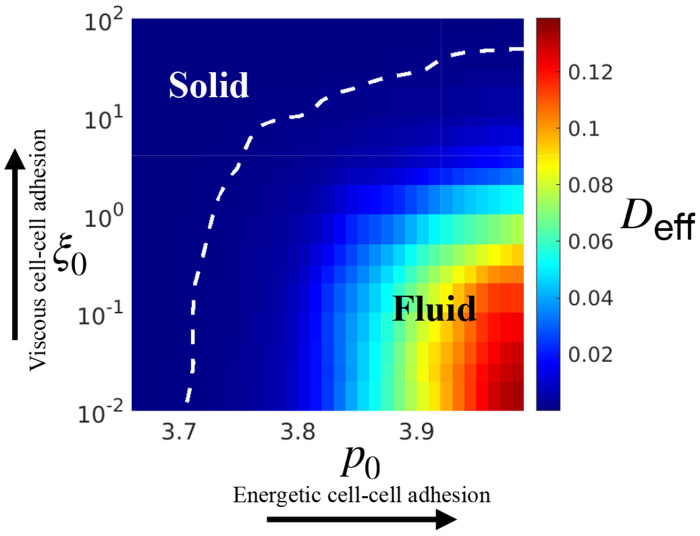
A jamming phase diagram governed by cell–cell adhesion. Tissue states are quantified using the effective diffusivity *D*_eff_, with the jamming–unjamming boundary defined by the equi-diffusivity contour *D*_eff_ = 0.001 (white dashed curve). The phase diagram is constructed from simulations with *v*_0_ = 0.05 and *D*_r_ = 0.5

The dual influence of cell–cell adhesion is summarized in the phase diagram shown in Fig. 3, where the effective diffusivity $D_{\text{eff}}$ serves as a dynamic order parameter. We classify states with $D_{\text{eff}}\leq 10^{-3}$ as jammed and those with $D_{\text{eff}}>10^{-3}$ as unjammed. This threshold correspond to the random noise floor in the simulation [31]. A corresponding jamming diagram constructed using $\tau_{\alpha }$ yields an essentially identical phase boundary, confirming the robustness of this classification (Fig. 7). A key result from the adhesion-driven phase diagram is the non-monotonic dependence of tissue fluidity on total cell–cell adhesion: increasing energetic adhesion promotes unjamming, whereas increasing viscous adhesion has the opposite effect and drives jamming. More broadly, this dual-role adhesion framework reconciles previously conflicting experimental reports on adhesion-mediated jamming [27, 28, 32] and underscores the importance of treating energetic and dissipative contributions as distinct, co-regulating factors in tissue mechanics.

### D. The role of cell-cell adhesion on tissue monolayer linear rheology

To better understand the viscoelastic property of the vertex model tissue, we analyze the tissue linear rheology. While the effect of the energetic adhesion parameter *p*_0_ on the storage and loss moduli of the vertex model has been studied previously [40], the effect of the kinetic adhesion parameter *ξ*_0_ on these quantities remain unexplored. To study the linear rheology of tissues, we performed strain-controlled simulations of the vertex model under oscillatory simple shear with the strain *γ* = *γ*_0_ sin(*ωt*). In our numerical simulations, we used *γ*_0_ = 0.001 and set *v*_0_ = 0. While recent analytical frameworks have begun to characterize the non-linear visco-elasto-plastic rheology of viscous vertex models under large deformations [57], our study deliberately focuses on the linear, small-strain regime to uncover the intrinsic spectrum of relaxation times. See Appendix for more details about the oscillatory deformation simulation protocol. After the system reached a steady state, we applied a Fourier transform to the tissue shear stress *σ*(*t*) and strain *γ*(*t*) to calculate the complex modulus G*. Details on the calculation of tissue shear stress and the complex modulus can be found in Appendix.

#### Oscillatory Rheology of Simulated Monolayers in the Fluid Regime

**FIG. 4.**
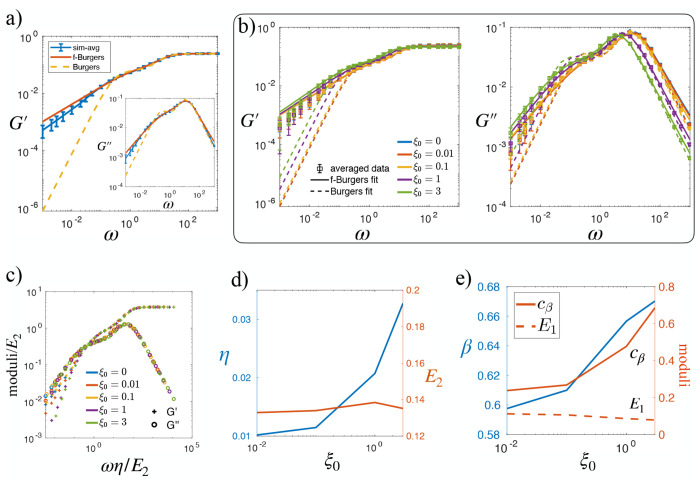
Linear rheology of model tissue in fluid regime at *p*_0_ = 3.9 suggests a broad spectrum of relaxation times: (a) The average complex modulus obtained from simulations at *ξ*_0_ = 0 was fitted to both the classical Burgers model and the fractional Burgers model. The curve represent the mean over eight independent initial conditions, and the error bars denote the standard deviation of *G*′ and *G*′′ at each frequency *ω*. (b) The complex modulus measured at different *ξ*_0_. Error bars indicate the mean ± standard deviation of *G*′ and *G*′′ at each *ω*, reflecting the variability across simulations. Solid and dashed lines correspond to fits using the fractional Burgers model and the classical Burgers model, respectively. (c) The collapse of different moduli curves for varying cell-cell adhesion *ξ*_0_. (d) Viscoelastic parameters of the conventional Maxwell branch in fractional Burgers model as a function of *ξ*_0_. (e) Viscoelastic parameters of the fractional Maxwell branch.

We first analyze the storage and loss moduli of the modeled tissues in the fluid-like state, with $p_{0}=3.9$ and varying $\xi_{0}$. Fig.8 shows the complex moduli for several single simulation at $\xi_{0}=0$. Interestingly, at low frequencies, the storage modulus $G^{\prime }$ does not scale as $\omega^{2}$, and the loss modulus does not scale as ω, as expected for a conventional viscoelastic fluid. Instead, both the storage and loss moduli scale as $\omega^{\beta }$ with 0<β<1, indicating a power-law behavior that suggests a broad spectrum of relaxation times. We examined eight different simulations, and this same power-law behavior persisted across all samples. The averaged complex moduli from these simulations are shown in Fig.4a, confirming the same scaling behavior. To highlight the difference between our modeled monolayer and a conventional viscoelastic fluid, we fit the simulated complex moduli to those predicted by the Burgers model using Eqn.15. As presented in Fig. 4(a), while the high-frequency behavior agrees with the Burgers model, clear deviations appear at low frequencies. To preserve the correct high-frequency trend while improving the fit at low frequencies, we replace one dashpot in the Burgers model with a springpot–a fractional viscoelastic element characterized by the constitutive relation $\sigma (t)=c_{\beta }\int_{0}^{t}{\left(t-\tau \right)}^{-\beta }\dot{\gamma }(\tau )d\tau$, thereby transforming the viscoelastic model into a fractional Burgers model. The analytical expression of the complex moduli of the fractional Burgers model is presented in Appendix. Fitting this fractional model (Eqn. 22) to our simulation data yields significantly improved agreement, particularly in the low-frequency regime (Fig.4a). Fig.4b further illustrates that the observed power-law behavior remains robust across different cell–cell adhesion strengths $\xi_{0}$.

To reveal the underlying universal viscoelastic response, we attempt to collapse the curves in Fig.4c by rescaling the moduli and frequency using the fitting parameters of the conventional Maxwell branch. Specifically, the moduli are rescaled by the elastic modulus $E_{2}$, while the frequency is rescaled by the reciprocal of the time constant $\eta /E_{2}$. As shown in Fig.4d, the curves collapse well in the high- and intermediate-frequency ranges, while slight deviations remain in the low-frequency regime, particularly for the storage modulus $G^{\prime }$. At high frequencies, the response is dominated by the elastic contribution of the springs, resulting in an effective collapse after rescaling. In the intermediate-frequency range, the peak of $G^{\prime \prime }$ and the onset of $G^{\prime }$ decay are governed by the viscous component η, which also allows the rescaled curves to align well. The residual spread in the low-frequency tails, however, suggests variations in the scaling exponent as $\xi_{0}$ increases. To further investigate this argument , we plot the fitting parameters of the fractional Burgers model as functions of $\xi_{0}$ in Fig.4e. In this panel, the role of $\xi_{0}$ in capturing the viscous contribution of adhesion forces is evident from the positive correlation between η and $\xi_{0}$. Moreover, $\xi_{0}$ also influences the springpot parameters—the exponent β and the amplitude $c_{\beta }$. In contrast, while $E_{1}$ and $E_{2}$ both decrease with increasing $p_{0}$, as reported previously [40], they remain insensitive to variations in $\xi_{0}$.

#### Oscillatory Rheology of Simulated Monolayers in the Solid Regime

**FIG. 5.**
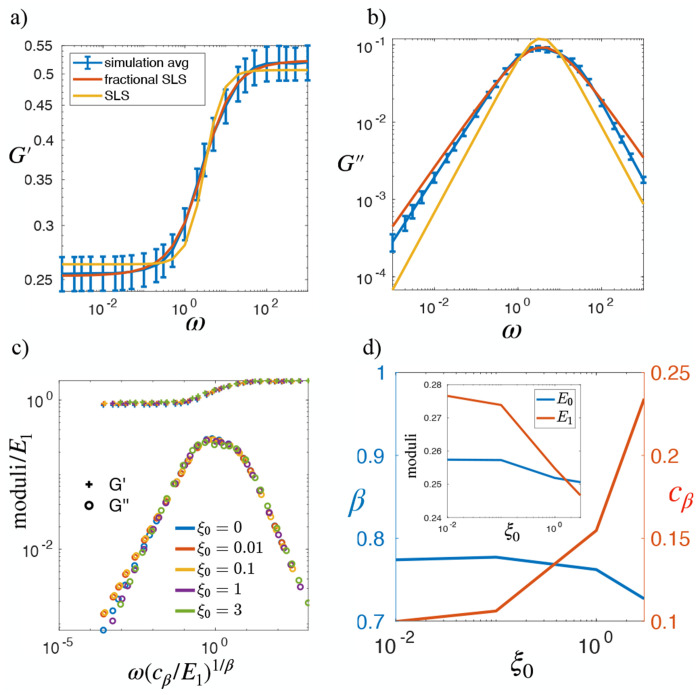
Linear rheology of model tissue in solid regime at *p*_0_ = 3.72 provides another evidence for the presence of multiple timescales in the system. (a,b) At *ξ*_0_ = 0, the average storage (*G*′) and loss (*G*′′) moduli are fitted using the fractional SLS and standard SLS models. The curves in panels (a) and (b) were obtained by averaging over eight different initial conditions; error bars represent the standard deviation. (c) Data collapse of different moduli curves for varying cell-cell adhesion *ξ*_0_. (d) Viscoelastic parameters as a function of *ξ*_0_.

To investigate the rheological response in the solid-jammed regime, we analyze the storage and loss moduli of modeled tissues at $p_{0}=3.72$ for varying $\xi_{0}$. Figures 5a,b show $G^{\prime }(\omega )$ and $G^{\prime \prime }(\omega )$ for $\xi_{0}=0$. At low frequencies, the storage modulus approaches a plateau, consistent with the response of monolayers in a solid-like regime. In contrast, the loss modulus $G^{\prime \prime }$ exhibits a power-law dependence $G^{\prime \prime }\propto \omega^{\beta }$ with 0<β<1 instead of scaling linearly with frequency ω. This fractional scaling persists across different $\xi_{0}$ values, as shown in Figure 9, highlighting a robust, time-scale–rich rheological behavior. To rationalize this observation, we compare the simulation results with both the Standard Linear Solid (SLS) model and its fractional generalization, in which we replace the dashpot by a springpot. The analytical forms for the complex moduli of the SLS model and fractional SLS model are presented in Eqn.13 and Eqn.24, respectively. As shown in Figure 5, the fractional SLS model captures the simulation data substantially better than the conventional SLS, particularly in reproducing the sub-linear power-law scaling of $G^{\prime \prime }$ at low frequencies. The superiority of the fractional model remains consistent across all tested $\xi_{0}$ values (Figure 9). Similar sublinear scaling of $G^{\prime \prime }$ with β<1 has been reported in concentrated oil-in-water emulsions and predicted in softsphere models, where it has been linked to the emergence of the Boson peak in the vibrational density of states [58].

To evaluate the ability of the fractional SLS model to capture the rheological behavior of monolayers in the solid regime, we attempt to collapse the complex moduli at different values of $\xi_{0}$ onto a single master curve. Unlike the fractional Burgers model—where the dashpot provides a natural relaxation timescale—the fractional SLS model contains no intrinsic characteristic time; its frequency dependence is controlled entirely by the springpot. Consequently, the appropriate rescaling must be constructed from the parameters of the fractional Maxwell branch. We therefore rescale the moduli by the elastic modulus $E_{1}$ and rescale the frequency by the springpot-derived timescale ${\left(E_{1}/c_{\beta }\right)}^{1/\beta }$, which follows directly from the dimensional structure of the fractional term. As shown in Fig. 5c, this rescaling produces an excellent collapse of the moduli across all tested $\xi_{0}$ values, in both the lowand high-frequency regimes, demonstrating that the response is governed by a common underlying scaling form. Notably, in contrast to the deviation between different rescaled $G^{\prime \prime }$ in the low frequency regime (Fig.4d), the solid-regime data collapse well, suggesting that the scaling exponent β is insensitive to $\xi_{0}$. This is confirmed by the fitted parameters of the fractional SLS model (Fig. 5d): the exponent β and the elastic modulus $E_{0}$ vary weakly with $\xi_{0}$. the springpot amplitude $c_{\beta }$ increases while the modulus $E_{1}$ decreases with increasing $\xi_{0}$, indicating that stronger cell–cell adhesion enhances the contribution of the springpot to the overall tissue rheology. Since the viscous response of the fractional SLS model used here arises solely from the springpot, the increase of $c_{\beta }$ with $\xi_{0}$ indicates that in solid-like regime, stronger cell–cell adhesion produces a larger effective viscosity, consistent with the role of $\xi_{0}$ as the parameter of junctional viscous dissipation in the vertex model. The robustness of the resulting sublinear power-law behavior in both the solid and fluid regimes suggests that a broad distribution of relaxation times is an intrinsic feature of tissue monolayers within the vertex-model framework. A detailed investigation into the origin of this multiscale relaxation structure lies beyond the scope of the present study and will be pursued in future work.

#### Stress Relaxation of Simulated Monolayers Under Step Strain

**FIG. 6.**
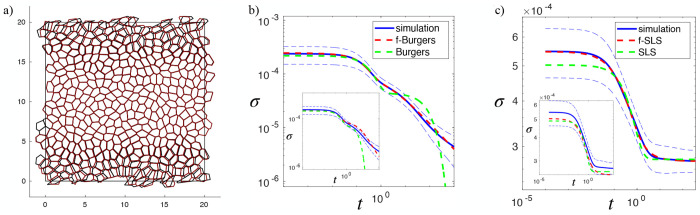
Relaxation modulus provides an independent confirmation of power-law rheology. a) Superimposed snapshots of the tissue configuration immediately after the application of a step strain and after long-time relaxation. Black polygons denote cell shapes directly following the imposed deformation, whereas red polygons correspond to the relaxed steady-state configuration. (b) Averaged stress relaxation after creep for monolayers at *p*_0_ = 3.9 and *ξ*_0_ = 1. Blue-thin dashed lines represent the variation in the stress between samples. The relaxation response is fitted using the fractional Burgers model and the conventional Burgers model. Inset: Relaxation moduli evaluated using parameters extracted from oscillatory simulations; the same legend applies. (c) Averaged stress relaxation following a step strain for monolayers at *p*_0_ = 3.72 and *ξ*_0_ = 1. Blue-thin dashed lines represent the variation in the stress between samples. The relaxation response is fitted using the fractional SLS model and the conventional SLS model. Inset: Relaxation moduli evaluated using parameters extracted from oscillatory simulations; the same legend applies.

**FIG. 7.**
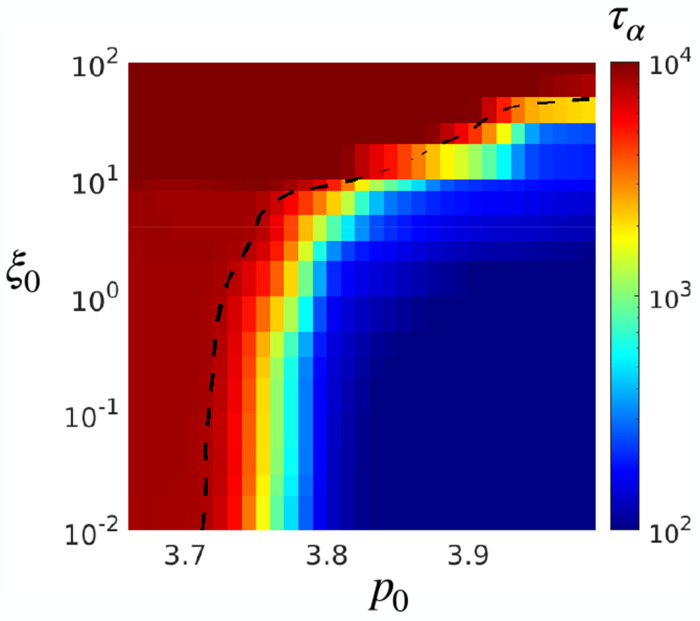
A second jamming phase diagram driven by cell–cell adhesion. Tissue states are quantified using the cell uncaging time $\tau_{\alpha }$, with the jamming-unjamming boundary defined by the contour $\tau_{\alpha }=156$. This threshold is selected so that the phase boundary at $\xi_{0}=0$ aligns with the boundary obtained from the diffusivity-based criterion.

**FIG. 8.**
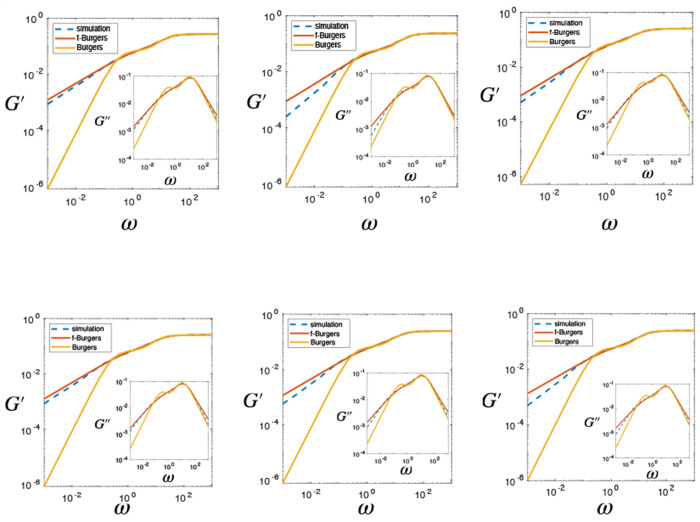
Complex moduli measured at *p*_0_ = 3.9 and *ξ*_0_ = 0 for multiple independent samples were fitted to both the classical Burgers model and the fractional Burgers model. The superior agreement with the fractional formulation demonstrates that the observed power-law rheology is intrinsic to the system and does not arise from sample-to-sample averaging.

**FIG. 9.**
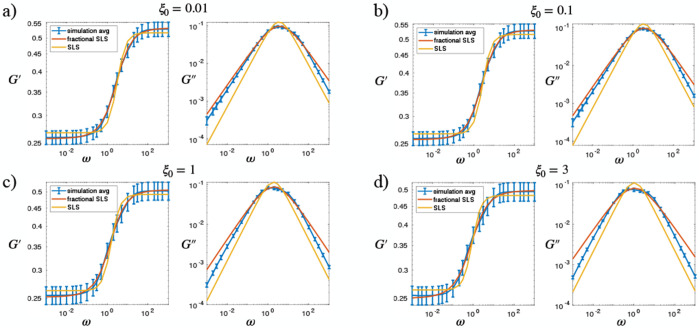
The storage and loss moduli at *p*_0_ = 3.72 across different values of *ξ*_0_, showing that the fractional SLS model consistently outperforms the conventional SLS model in capturing the frequency-dependent stress response.

Motivated by the low-frequency scaling of the complex moduli, we examine the relaxation modulus G(t) and expect a corresponding power-law decay. To test this prediction, we perform stress-relaxation simulations by imposing a small step strain of $\gamma_{0}=10^{-4}$ and holding it fixed while monitoring the resulting stress decay. To isolate the intrinsic material response, T1 transitions are prohibited during relaxation. The resulting curves, $\sigma (t)=\gamma_{0}G(t)$, for monolayers with $p_{0}=3.9$ and 3.72 at $\xi_{0}=1$, are shown in Fig. 6a and b, respectively. As anticipated, the relaxation modulus at $p_{0}=3.9$ displays a power-law decay, consistent with a fluid-like material lacking a single characteristic relaxation timescale. Power-law relaxation in biological system has been reported at both the cellular scale [59] and the tissue scale [38, 39]. Fitting these curves to the Burgers and fractional Burgers models shows that the fractional model yields a substantially improved description (Fig. 6a), a result that holds across all tested values of $\xi_{0}$. Because a power-law decay in G(t) implies a power-law growth in the creep compliance with the same exponent β, this observation aligns with recent experiments on Drosophila embryonic epithelium. Using ferro-fluid droplets under a constant pulling force, the displacement versus time relation of these droplet while inside tissues capture the creep compliance of the tissue, with a scaling law of exponent β≈1/2 has been reported [60].

In the solid regime, the fractional SLS model likewise provides a superior fit to the simulated step-strain response relative to the conventional SLS model (Fig. 6b). The improved performance of the fractional viscoelastic models in both fluid-like and solid-like regimes further supports the interpretation that tissue monolayers possess a broad distribution of relaxation times. We also use the fitting parameters obtained from oscillatory measurements to predict the stress-relaxation response (insets of Fig. 6a,b). Although these predictions do not perfectly reproduce the relaxation curves, they remain considerably more accurate than predictions from the corresponding conventional models. The discrepancies between parameters extracted from oscillatory and relaxation protocols are unsurprising. In the oscillatory simulations, the continuous cyclic driving effectively mechanically anneals the tissue: repeated oscillations progressively relax internal stresses, guide the system toward a smoother energy landscape, and place it in a more homogeneous, well-annealed mechanical state. In contrast, the relaxation simulations begin only from an instantaneously equilibrated configuration, which is equilibrated in the sense of mechanical force balance but is not necessarily stress-free and may retain residual disorder inherited from the initial condition. Because no mechanical cycling occurs during relaxation, the system remains in this comparatively poorly annealed state throughout the step-strain test. Consequently, the material parameters inferred from relaxation data reflect these less-annealed configurations, whereas the oscillatory measurements probe a more mechanically conditioned ones.

## III. DISCUSSION

In this work, we addressed longstanding paradox regarding the role of cell–cell adhesion in tissue dynamics, which can be characterized using either the effective diffusivity *D*_eff_ or the uncaging relaxation time *τ_α_*, both of which are experimentally accessible measures, and we examined how adhesion regulates the viscoelastic response of epithelial monolayers within the vertex-model framework. Our approach explicitly disentangles two mechanistically distinct components of adhesion: an energetic contribution, which reduces junctional tension, and a kinetic contribution, which resists relative motion across the shared cell–cell interface. The latter is incorporated into the vertex model with support from a microscopic description showing that relative motion between adjacent cell surfaces, together with adhesion-bond forces, generates an effective mesoscopic drag. We demonstrated that increasing the kinetic component of adhesion progressively enhances dynamical arrest, ultimately driving the system into a jammed state. However, this jamming effect competes with the fluidization induced by the energetic adhesion component, which has been extensively characterized [30, 31]. As a result of this competition, the tissue exhibits a non-monotonic dependence on adhesion strength, such that increasing adhesion can initially promote unjamming before leading to jamming at higher values. The corresponding phase diagram captures this complex, non-monotonic jamming–unjamming behavior and delineates the regimes in which either mechanism dominates. By capturing the competing effects of energetic and kinetic adhesion, our framework resolves previously contradictory experimental findings on adhesion-driven jamming in biological tissues [22, 27, 28, 32] and provides a unified perspective on how adhesion modulates cell motion and tissue dynamics.

We further investigated how cell–cell adhesion influences the rheological properties of vertex-model monolayers subjected to both oscillatory and step-strain deformation. The resulting stress responses in each case exhibit power-law behavior, motivating their analysis using fractional viscoelastic models. The power-law behavior reported here further suggests that rich, multiscale relaxation dynamics can emerge even within a minimal framework such as the vertex model. This behavior becomes more pronounced when the response is averaged over multiple realizations. Because the model is self-averaging—so that ensemble averaging over samples is equivalent to spatial averaging over different regions of the monolayer—the enhanced prominence of the power law suggests that the vertex-model tissue is intrinsically heterogeneous in its local relaxation timescales. A systematic finite-size analysis of this heterogeneity, including the distribution and spatial organization of local relaxation times, would help identify the microscopic origin of the observed multiscale behavior and further elucidate why a broad spectrum of relaxation processes is an inherent feature of tissue monolayers within the vertex-model framework. More broadly, the linear rheological response observed in our simulations reinforces the view that epithelial tissues behave as viscoelastic materials with non-exponential, scale-free relaxation. Analysis within the extended vertex model indicates that, by modulating both elastic storage and viscous dissipation, cell–cell adhesion emerges as a key regulator of epithelial monolayer viscoelasticity and dynamical state, with the capacity to soften the tissue while simultaneously enhancing its viscous properties. It is important to note that the absolute glassy arrest observed in our framework is inherently tied to the isotropic active driving and 2D topological constraints of the standard vertex model. For instance, recent multiphase field models demonstrate that when motility is coupled with intercellular friction, the resulting spatial flow correlations can drive macroscopic orientational order rather than simple arrest [61]. Furthermore, 3D active foam models suggest that out-of-plane degrees of freedom and continuous cell-cell debonding may prevent the tissue from fully solidifying even under high intercellular friction [62]. Reconciling these dimensionality and hydrodynamic effects with our dual-role adhesion framework represents an important direction for future research.

### Quantitative Predictions for Experiments

**FIG. 10.**
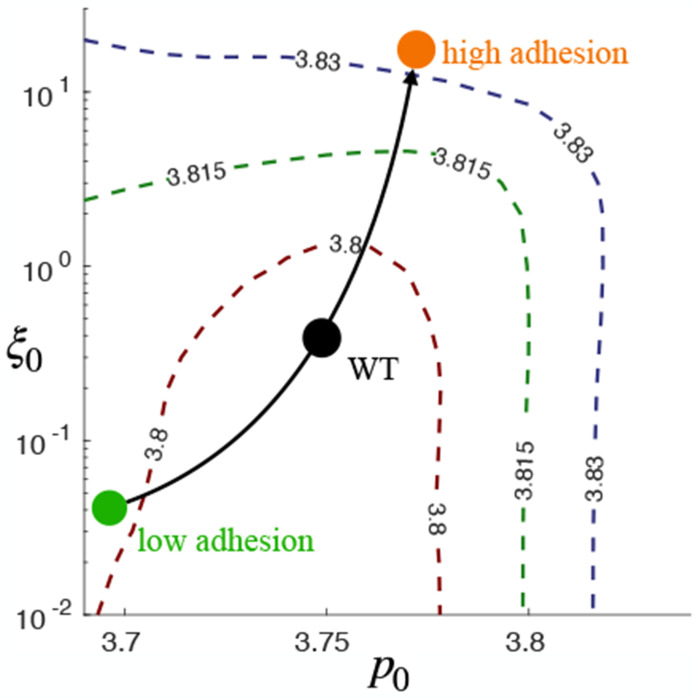
A hypothetical trajectory in the phase diagram illustrating the non-monotonic dependence of cell shape on adhesion strength. Along a path of increasing adhesion—represented here by a curve with positive slope—the cell shape may first decrease and subsequently increase, consistent with observations in the Drosophila embryo.

To illustrate the predictive scope of the extended vertex model, we apply it to several experimentally reported phenomena. In particular, the dual-role formulation of adhesion provides a unified interpretation of the non-monotonic dependence of cell shape on cell–cell adhesion level—quantified by junctional E-cadherin intensity—in the Drosophila embryo during axis elongation [37]. In that study, one perturbation reduced adhesion and another increased it relative to wild type, yet both conditions exhibited elevated cell shape indices compared to wild type. Within our framework, this seemingly paradoxical behavior arises because adhesion modulates tissue mechanics through separable energetic and dissipative contributions. Changes in adhesion can therefore shift the system through distinct regions of the phase diagram without requiring a monotonic geometric response. A representative trajectory capturing this qualitative behavior is shown in Fig. 10.

**FIG. 11.**
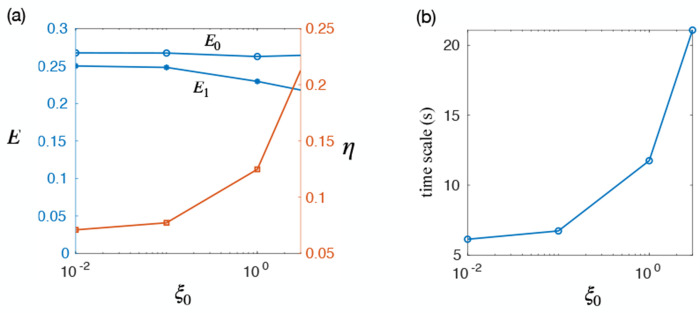
Extraction of the relaxation timescale from SLS model fits.(a) Standard Linear Solid (SLS) fitting parameters as a function of viscous adhesion *ξ*_0_. (b) Using the time scale calibration reported in [36], the relaxation timescale *τ* = *η/E*_1_ is converted into physical time units, yielding values consistent with experimentally measured stress relaxation times in microtissues [63].

We further connect the rheological predictions of the extended vertex model to experimental measurements. Specifically, we compared the relaxation timescales obtained in simulations with measurements in microtissues reported by Walker et al. [63]. In those experiments, microtissues were subjected to step-strain deformations using cantilevers and allowed to relax under fixed strain. Using the fitted parameters of the Standard Linear Solid (SLS) model in the solid regime (Fig. 11) together with the time calibration reported in [36], we converted the characteristic relaxation time *τ* = *η/E*_1_ into physical units. The resulting relaxation times, which vary with *ξ*_0_ and lie in the range of approximately 6–20 seconds, are comparable to the experimentally reported value of roughly 14 seconds. This level of agreement supports the physical interpretability of the model parameters and suggests that the emergent rheological timescales are within a quantitatively reasonable range.

Additionally, the dependence of the rheological parameters *β*, *c_β_*, and *η* on the viscous adhesion coefficient *ξ*_0_, shown in Fig. 4, establishes a direct link between vertex-model predictions and observations in MDCK monolayers treated with the Y27632 ROCK inhibitor, a biochemical perturbation known to substantially increase cell–cell adhesion [64, 65]. In those simulations, the relaxation modulus *G*(*t*) was analyzed using a fractional viscoelastic model analogous to our fractional Burgers formulation, and Y27632 treatment produced a marked increase—approximately two-fold—in the effective viscosity *η*, accompanied by a reduction in the elastic modulus *E*_2_ [39]. While the adhesion-induced softening is consistent with predictions from the conventional vertex model [40], the concomitant increase in viscosity is not captured within that framework. By contrast, the extended model naturally reproduces this effect through the explicit dependence of both the springpot amplitude *c_β_* and the viscosity *η* on *ξ*_0_. This result reinforces the conclusion that viscous dissipation at cell–cell junctions is a central determinant of epithelial monolayer rheology within the vertex-model description.

The examples presented are preliminary demonstrations of the model’s scope rather than a systematic or exhaustive mapping of parameter space to experimental observables. A more comprehensive and quantitatively rigorous comparison with experiments is presented in the companion study [36]. In those experiments, cell–cell adhesion was perturbed while other major regulators of tissue fluidity were held effectively constant, thereby isolating adhesion as the principal control parameter of tissue fluidity. Under these conditions, we observed a pronounced enhancement of tissue fluidity that occurred without a corresponding change in cell shape index. This shape-independent fluidization cannot be reconciled with the original vertex model, in which geometry serves as the primary order parameter for rigidity. In contrast, the extended model captures this behavior by incorporating viscous adhesion as an independent mechanical contribution. The observation that tissue fluidity can be modulated independently of cell shape challenges the prevailing geometric paradigm and motivates a broader framework in which dissipative mechanisms play a central role in governing epithelial dynamics.

### Outlook

In many experimental studies of tissue jamming and unjamming [7, 27, 28, 32, 47], cell–substrate mechanical interactions play a central role in addition to cell–cell adhesion. Traction forces, substrate stiffness, and active motility collectively influence stress generation, energy dissipation, and collective rearrangements. Within the vertex-model framework, these active effects are commonly coarse-grained into the motility parameter *v*_0_, while dissipative interactions with the substrate are grounded by a baseline friction coefficient (*μ*). The present work isolates the contribution of cell–cell adhesion in order to clarify its dual energetic and dissipative roles. However, future extensions of this model could comprehensively explore the full spectrum of cell–substrate coupling. On one end, incorporating the effect of v0 would naturally extend the current adhesion-controlled phase diagram into a three-dimensional jamming landscape, enabling systematic exploration of how active driving and intercellular coupling cooperate to regulate yielding and collective flow. On the opposite extreme, to investigate completely free-floating epithelia, our dual-role adhesion framework could be integrated with recent theoretical advances that formulate rotationally invariant viscous vertex matrices capable of simulating zero-substrate-friction dynamics [66].

## Figures and Tables

**FIG. 1.**
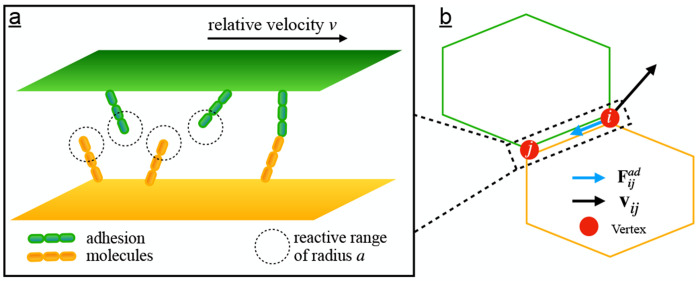
Modeling cell-cell adhesion force in vertex model. a) A microscopic model of cell-cell adhesion force. b) Schematic of adhesion force implementation in vertex model

## IV. APPENDIX

### A. Solving the equation of motion

Due to the adhesion force implemented, the *x* and *y* directions are coupled. The equation of motion can be rewritten in matrix form as:

(5)
Mv=f

For a system of size N,f is a 4N×1 column vector represents the interaction force on each vertices, with the first 2N entries are the x-component of the forces and the remaining 2N entries are the y-component of the forces. Similarly, v is a 4N×1 column vector storing the velocities of the vertices. **M** is a 4N×4N sparse matrix, which can be expressed as:

$$M=\left[\begin{array}{ll} M^{xx} & M^{yx}\\ M^{xy} & M^{yy} \end{array}\right]$$

Where $M^{\alpha \beta }$, with α,β∈{x,y}, is a 2N×2N matrix encoding the coupling between the velocities in the α-direction and the force in the β-direction. The non-zero elements in those sub-matrices are $M_{ii}^{\alpha \beta }=\delta_{\alpha ,\beta }+\xi_{0}\sum_{j\in S_{i}}r_{ij}^{\alpha }r_{ij}^{\beta }/\left|r_{ij}\right|$ and $M_{ij}^{\alpha \beta }=-\xi_{0}r_{ij}^{\alpha }r_{ij}^{\beta }/\left|r_{ij}\right|$ for $j\in S_{i}.M^{\alpha \beta }$ can therefore be conveniently constructed from the 4N×1 vertex position vector r(t) and the vertex-vertex adjacency matrix. The equation of motion can be integrated numerically as

(6)
$$r(t+\Delta t)=r(t)+\Delta tM^{-1}f(t)$$

### B. The stress tensor for each cell in the presence of cell-cell adhesion force

In our model, the stress tensor $\sigma_{c}$ for cell c is expressed as:

(7)
$$\sigma_{c}=\sigma_{c}^{int}+\sigma_{c}^{ad}$$

where $\sigma_{c}^{\mathrm{int}}$ is the stress tensor due to the interaction force, which can be computed as [67]:

(8)
$$\sigma_{c}^{int}=K_{A}\left(A_{c}-A_{0}\right)I+\frac{1}{A_{c}}\sum_{e\in c}K_{P}\left(P_{C}-P_{0}\right){\hat{L}}_{e}⊗L_{e}$$

where I is the unit tensor, $L_{e}$ is edge e vector, with e∈c represents the set of edges of cell c, and ⊗ is the tensor product notation. $\sigma_{c}^{ad}$ is the stress tensor due to the adhesion force, which can be computed as[68]:

(9)
$$\sigma_{c}^{ad}=\sum_{i\in c}=-\frac{1}{2z_{c}A_{c}}\sum_{i\in c}\left(R_{i}⊗F_{i}^{ad}+F_{i}^{ad}⊗R_{i}\right)$$

where $z_{c}$ is the number of vertices cell c has, $R_{i}=r_{i}-r_{c}$ is the position of vertex i relative to cell c, and $F_{i}^{ad}$ is the total adhesion force at vertex i. The tissue stress tensor σ was then computed as:

(10)
$$\sigma =\frac{\sum_{c}A_{c}\sigma_{c}}{\sum_{c}A_{c}}$$

The tissue shear stress was then extracted from the tissue stress tensor as $\sigma =\sigma_{xy}$.

### C. Oscillatory and stepwise shear for tissue linear response rheology

To examine the mechanical behavior of the tissue, we impose oscillatory simple shear deformation on the simulated system using Lees-Edwards boundary conditions[69]. Initially, strain-free configurations were generated and then relaxed according to Eqn 3 with ξ=0 and $v_{0}=0$ using FIRE algorithm[70]. The affine deformation was subsequently applied according to the deformation gradient tensor $D=\left(\begin{array}{cc} 1 & \gamma_{0}sin\;(\omega t)\\ 0 & 1 \end{array}\right)$. Following the affine deformation at every time step, the system was updated based on the overdamped dynamics described by Eqn. 3 with $v_{0}=0$ using Euler method. As we focus on the viscoelastic properties of tissues, T1 transitions were disabled during the oscillatory deformation process.

To ensure that the system reaches steady state, after each deformation cycle q of period $T=2\pi /\omega_{0}$ for a particular driving frequency $\omega_{0}$, we compute the Fourier transform of the stress in the most recent 5 cycle:

(11)
$${\tilde{\sigma }}_{q}\left(\omega_{0}\right)=\frac{1}{5T}\int_{(k-5)T}^{kT}\sigma (t)e^{i\omega_{0}t}dt$$

The system was considered to have reached steady state once ${\tilde{\sigma }}_{q}\left(\omega_{0}\right)$ stabilized and no longer changed significantly with additional cycles, approaching a consistent value denoted by $\tilde{\sigma }\left(\omega_{0}\right)$. The complex shear modulus was then calculated as $G^{*}\left(\omega_{0}\right)=\tilde{\sigma }\left(\omega_{0}\right)/\tilde{\gamma }\left(\omega_{0}\right)$, where $\tilde{\gamma }\left(\omega_{0}\right)$ is the Fourier transform of the applied strain γ(t).

To perform the numerical relaxation experiment, we applied a step strain to the system, holding the strain and let the system relax. The corresponding deformation gradient tensor for this deformation is $D=\left(\begin{array}{cc} 1 & \gamma_{0}ℋ(t)\\ 0 & 1 \end{array}\right)$, where ℋ(t) is the Heaviside function.

### D. Linear Rheological models

#### 1. The Standard Linear Solid (SLS) model

The Standard Linear Solid (SLS) model considered in this work consists of a spring with spring constant *E*_0_ in parallel with a Maxwell component of elasticity *E*_1_ and viscosity *η*. Since the material is composed from two parallel branches, the modulus is the sum of the modulus of the two branches, giving the relaxation modulus:

(12)
$$G_{SLS}(t)=E_{0}+E_{1}e^{-\frac{tE_{1}}{\eta }}$$

and the complex modulus of the SLS material:

(13)
$$G_{SLS}^{*}(\omega )=E_{0}+\frac{i\omega E_{1}\eta }{E_{1}+i\omega \eta }$$

#### 2. The Burgers model

The Burgers model considered here consists of two Maxwell components ($E_{1},\eta_{1},E_{2},\eta_{2}$) in parallel. The relaxation modulus of this Burgers model is:

(14)
$$G_{\text{Burgers}}(t)=E_{1}e^{-tE_{1}/\eta_{1}}+E_{2}e^{-tE_{2}/\eta_{2}}$$

and the complex modulus is:

(15)
$$G_{\text{Burgers}}^{*}(\omega )=\frac{i\omega E_{1}\eta_{1}}{E_{1}+i\omega \eta_{1}}+\frac{i\omega E_{2}\eta_{2}}{E_{2}+i\omega \eta_{2}}$$

#### 3. The Fractional Burgers model

Our Fractional Burgers model consists of a Maxwell component in parallel with a fractional Maxwell component. The fractional Maxwell component consists of a spring $E_{1}$ in series with a springpot with exponent β and strength $c_{\beta }$. The creep compliance of the springpot is $J_{\beta }(t)=\frac{t^{\beta }}{c_{\beta }\Gamma (1+\beta )}$ [71]. Therefore, the creep compliance of the fractional Maxwell material is:

(16)
$$J(t)=\frac{1}{E_{1}}+\frac{t^{\beta }}{c_{\beta }\Gamma (1+\beta )}$$

The Laplace transform of the creep compliance is:

(17)
$$J(s)=\frac{1}{E_{1}s}+\frac{1}{c_{\beta }s^{\beta +1}}$$

Using the fundamental relation between relaxation modulus and creep compliance, $G(s)J(s)=1/s^{2}$ [72], the Laplace transform of the relaxation modulus of the fractional Maxwell material can be obtained:

(18)
$$G_{\text{f-Maxwell}}(s)=\frac{1}{J(s)s^{2}}=\frac{E_{1}s^{\beta -1}}{s^{\beta }+E_{1}/c_{\beta }}$$

The relaxation modulus can then be obtained by taking the inverse Laplace transform of G(s), exploiting the integral representation of the Mittag-Leffler function [73]:

(19)
$$G_{\text{f-Maxwell}}(t)=E_{1}{ℳ}_{\beta ,1}(-\frac{E_{1}}{c_{\beta }}t^{\beta })$$

where ${ℳ}_{a,b}$ is the Mittag–Leffler function with parameters a and b. The relaxation modulus of a fractional Burgers model can then be obtained by adding the contribution of the conventional Maxwell branch to the modulus[74]:

(20)
$$G_{\text{f-Burger}}(t)=E_{1}{ℳ}_{\beta ,1}(-\frac{E_{1}}{c_{\beta }}t^{\beta })+E_{2}e^{-tE_{2}/\eta }$$

To obtain the complex modulus of the Fractional Maxwell model, we exploit the linear rheological assumption that under oscillatory strain $\gamma =e^{i\omega t}$, the stress is also harmonic $\sigma =G^{*}e^{i\omega t}$. This assumption can be combined with the constitutive equation $\gamma (t)=\frac{\sigma (t)}{E_{1}}+\frac{\sigma (t)t^{\beta }}{c\beta \Gamma (1+\beta )}$ to obtain the complex modulus of the fractional Maxwell branch:

(21)
$$G_{f-Maxwell}^{*}(\omega )=\frac{E_{1}c\beta (i\omega {)}^{\beta }}{E_{1}+c_{\beta }(i\omega {)}^{\beta }}$$

The complex modulus of the Burgers model therefore is[74]:

(22)
$$G_{\text{f-Burger}}^{*}(\omega )=\frac{E_{1}c\beta (i\omega {)}^{\beta }}{E_{1}+c_{\beta }(i\omega {)}^{\beta }}+\frac{i\omega E_{2}\eta }{E_{2}+i\omega \eta }$$

#### 4. The Fractional SLS model

In our Fractional SLS model, the conventional Maxwell branch is replaced by a fractional Maxwell component. Using Eqn 19, the relaxation modulus can be obtained as:

(23)
$$G_{f-SLS}(t)=E_{0}+E_{1}{ℳ}_{\beta ,1}\left(-\frac{E_{1}}{c_{\beta }}t^{\beta }\right)$$

Similarly, using Eqn 21, the complex modulus of the fractional SLS is:

(24)
$$G_{f-SLS}^{*}(\omega )=E_{0}+\frac{E_{1}c_{\beta }(i\omega {)}^{\beta }}{E_{1}+c_{\beta }(i\omega {)}^{\beta }}$$
